# Fluid Overload-Associated Large B-Cell Lymphoma: A Case Report and Review of Literature

**DOI:** 10.3390/hematolrep15030042

**Published:** 2023-07-03

**Authors:** Hisham F. Bahmad, Aaron S. Gomez, Arunima Deb, Fernando Martin Safdie, Vathany Sriganeshan

**Affiliations:** 1 The Arkadi M. Rywlin M.D. Department of Pathology and Laboratory Medicine, Mount Sinai Medical Center, Miami Beach, FL 33140, USA; arunima.deb@msmc.com (A.D.); vathany.sriganeshan@msmc.com (V.S.); 2 Department of Pathology, Herbert Wertheim College of Medicine, Florida International University, Miami, FL 33199, USA; agome399@med.fiu.edu; 3 Department of Surgery, Division of Thoracic and Cardiovascular Surgery, Mount Sinai Medical Center, Miami Beach, FL 33140, USA; fernando.safdie@msmc.com

**Keywords:** fluid overload-associated large B-cell lymphoma, pleural effusion, primary effusion lymphoma, large B-cell lymphoma, case report

## Abstract

Fluid overload-associated large B-cell lymphoma (FO-LBCL) is a new entity described in the fifth edition of the World Health Organization (WHO) Classification of Hematolymphoid Tumors (WHO-HAEM5). It refers to malignant lymphoma present with symptoms of serous effusions in body cavities (pleural, peritoneal, and/or pericardial) in the absence of an identifiable tumor mass. We present a case of an 82-year-old man with a history of atrial fibrillation and atrial flutter, status post-ablation, essential hypertension (HTN), hyperlipidemia (HLD), and diabetes mellitus (DM) type 2 who was referred to our hospital for shortness of breath due to recurrent pleural effusion. Right video-assisted thoracoscopy with right pleural biopsy was performed. Histopathological examination of the pleural biopsy revealed dense fibrous tissue, chronic inflammation, lymphoid aggregates, and granulation tissue, with no evidence of lymphoma. Cytology of the right pleural fluid revealed large lymphoid cells, which were positive for CD45, CD20, PAX-5, MUM-1, BCL2, BCL6, and MYC protein. They were negative for CD3, CD10, CD138, and HHV-8 by immunohistochemistry (IHC). Epstein–Barr virus (EBV) was negative by in situ hybridization (ISH). Due to the absence of any evidence of lymphoma elsewhere, a diagnosis of fluid overload-associated large B-cell lymphoma (FO-LBCL) was made. We provide a synopsis of the main clinicopathological features of FO-LBCL and the two main differential diagnoses, primary effusion lymphoma (PEL) and diffuse large B-cell lymphoma (DLBCL).

## 1. Introduction

Fluid overload-associated large B-cell lymphoma (FO-LBCL) refers to a new entity described in the fifth edition of the World Health Organization (WHO) Classification of Hematolymphoid Tumors (WHO-HAEM5) [1]. There are rare cases where patients with malignant lymphomas present with symptoms of serous effusions in body cavities in the absence of an identifiable tumor mass [2]. Historically, this presentation has been associated with primary effusion lymphoma (PEL), a human herpesvirus 8 (HHV8)-positive B-cell lymphoma with a predilection for patients that are immunocompromised, typically in the setting of human immunodeficiency virus (HIV) infection [2]. However, cases of effusion-based lymphomas have been reported which are not only HHV8-negative, but also manifest in a different patient population than those seen with PEL [3].

Terms such as primary effusion lymphoma, HHV8-negative PEL, or HHV8-unrelated PEL-like lymphoma have been used previously to identify this entity [4]. However, given the commonality of a fluid overload state as a comorbidity among these reported cases, fluid-overload effusion lymphoma along with Kaposi sarcoma-associated herpes virus (KSHV)/HHV8-negative effusion-based lymphoma have become acceptable terminology to refer to this entity, distinguishing them from PEL [5]. FO-LBCL previously fell under the umbrella of diffuse large B-cell lymphoma with chronic inflammation in the revised fourth edition of the WHO Classification of Hematolymphoid Tumors (WHO-HAEM4). However, the WHO-HAEM5 categorizes FO-LBCL as a rare subtype of large B-cell lymphoma with a specific clinicopathological presentation [1].

We present a case of an 82-year-old man with recurrent right pleural effusions. Pleural biopsy showed dense fibrous tissue, chronic inflammation, and lymphoid aggregates supportive of a reactive process, whereas the pleural fluid cytology specimen demonstrated large B-cell lymphoma supported by immunohistochemical (IHC) stains. This case report was conducted and reported in accordance with the Case Reports (CARE) guidelines for reporting case reports.

## 2. Case Presentation

An 82-year-old man with a history of atrial fibrillation and atrial flutter status post-ablation, essential hypertension (HTN), hyperlipidemia (HLD), and diabetes mellitus (DM) type 2 was referred to our hospital for shortness of breath due to recurrent pleural effusion. The patient denied cough, chest pain, or fever. He also had coronary artery disease (CAD), for which he underwent coronary artery bypass graft (CABG) surgery a few years ago. Surgical history was significant for coronary angioplasty with stent placement and CABG in 2016. No relevant family history was present.

Upon presentation, the patient’s vital signs were as follows: arterial blood pressure was 156/78 mmHg, pulse was 70 beats per minute, respiratory rate was 16 per minute, temperature was 36.3 °C, and SpO_2_ was 97%. Body mass index (BMI) was 29.54 kg/m^2^ (weight 83 kg and height 167.6 cm). Physical examination was significant for lower extremity swelling. Laboratory results are summarized in Table 1. The patient had microcytic anemia and thrombocytopenia; the thrombocytopenia appeared to be persistent since 2016. Electrolyte levels and urinalysis were within normal limits. The patient had no history of hepatitis C virus (HCV) infection.

**Figure 1:**
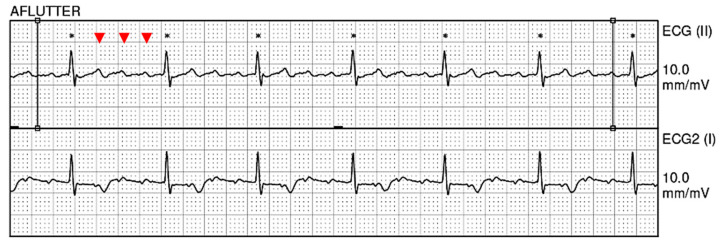
A 12-lead electrocardiogram (EKG) showing atrial flutter (red arrows) with 4:1 A-V conduction.

**Figure 2:**
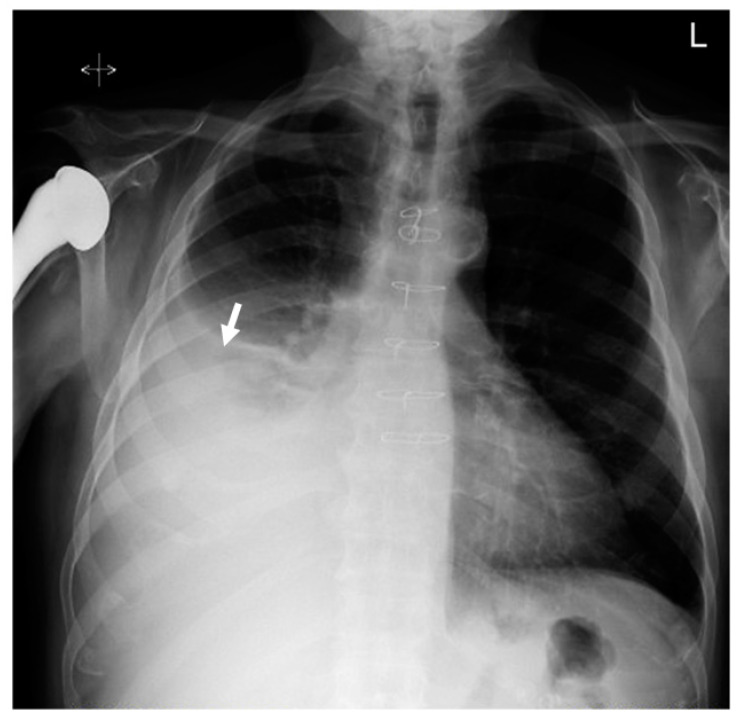
Chest X-ray demonstrating stable large right-sided pleural effusion (white arrow) with associated atelectasis/consolidation.

**Figure 3:**
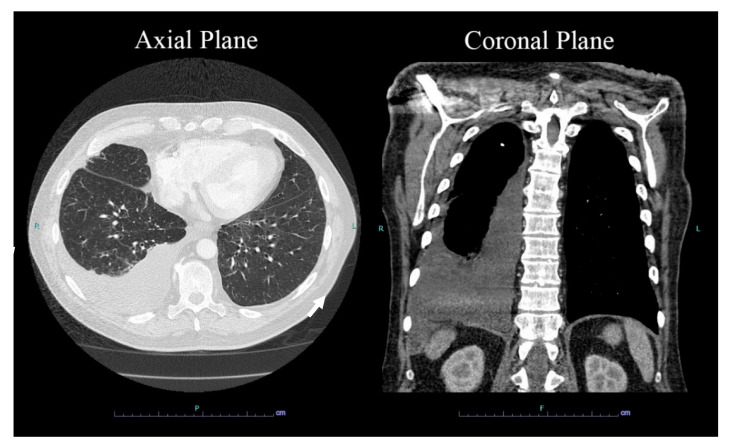
Imaging studies. Axial plane (**left panel**) and coronal plane (**right panel**) computed tomography (CT) scan with IV contrast of the chest showing moderate right pleural effusion (white arrows) with a small amount of compressive airspace disease at the right base.

An echocardiogram showed normal left ventricular systolic function with a left ventricular ejection fraction (LVEF) between 60 and 65%. The patient had trace aortic regurgitation and mild mitral regurgitation. Trace tricuspid regurgitation was also noted. A 12-lead electrocardiogram (EKG) revealed atrial flutter with 4:1 A-V conduction in addition to nonspecific ST and T wave abnormalities, suggestive of remote lateral ischemia (Figure 1). Chest X-ray demonstrated stable large right-sided pleural effusion with associated atelectasis/consolidation, a cardio-mediastinal silhouette within normal limits, intact median sternotomy wires, and aortic arch calcifications (Figure 2). A computed tomography (CT) chest scan showed moderate right pleural effusion with a small amount of compressive airspace disease at the right base (Figure 3).

**Figure 4:**
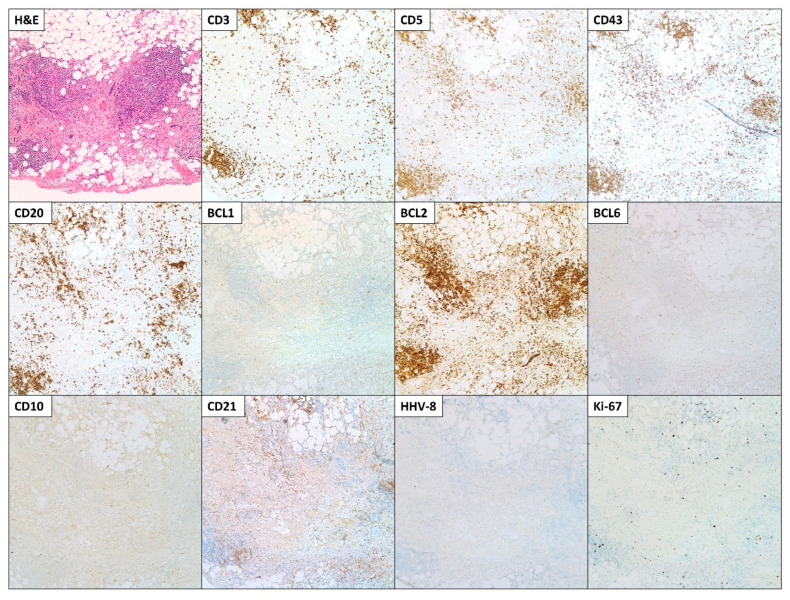
Microscopic images of the hematoxylin and eosin (H&E) and immunohistochemical (IHC) stains (performed using a blue stain (hematoxylin) as background color and a brown stain (diaminobenzidine; DAB) to reveal positivity) of the pleural biopsy. The pleural biopsy showed dense fibrous tissue, chronic inflammation, lymphoid aggregates, and granulation tissue. IHC stains were positive for CD3, CD5, CD43, and CD20, showing a mixed population of T and B lymphocytes. CD10 and BCL1 were negative, while BCL6 was focally positive and BCL2 was positive, predominantly in the T lymphocytes. CD21 was positive, highlighting small follicular dendritic networks within the lymphoid follicles. HHV-8 was negative. Ki-67 was positive with a low proliferation rate (<10%). Microscopic images were examined at 200× objective.

Right video-assisted thoracoscopy with right pleural biopsy, Talc pleurodesis, and indwelling tunnel pleural catheter placement was performed, draining 2300 mL of serosanguinous to bloody fluid. Cytology specimen as well as the right pleural biopsy specimens were sent to pathology. The pleural biopsy showed dense fibrous tissue, chronic inflammation, lymphoid aggregates, and granulation tissue (Figure 4). Immunohistochemical stains for CD3, CD5, and CD43 (T-cell associated markers), as well as CD20 (B-cell associated marker), were positive, showing a mixed population of T and B lymphocytes. CD10 was negative while BCL6 (germinal center marker) was focally positive in the follicles. BCL2 was positive, predominantly in the T lymphocytes. BCL1 was negative. CD21 was positive, highlighting small follicular dendritic networks within the lymphoid follicles. HHV-8 was negative. Ki-67 was positive with a low proliferation rate (<10%). These findings supported a reactive process.

**Figure 5:**
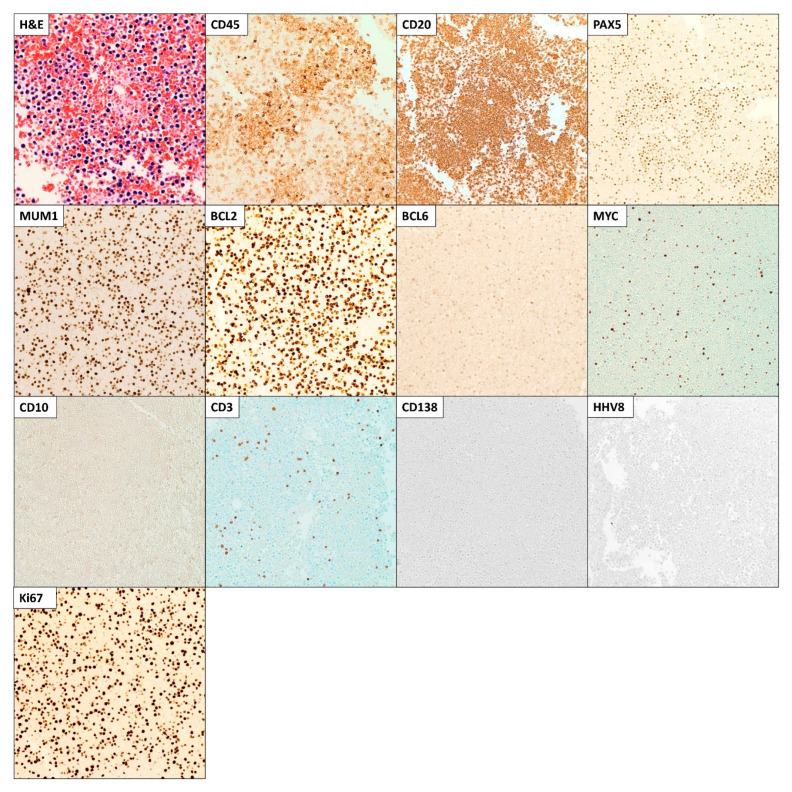
Microscopic images of the hematoxylin and eosin (H&E) and immunohistochemical (IHC) stains (performed using a blue stain (hematoxylin) as background color and a brown stain (diaminobenzidine; DAB) to reveal positivity) of the right pleural fluid cytology specimen. Large atypical lymphoid cells with irregular nuclei, prominent nucleoli, coarse chromatin, and moderate cytoplasm were seen. The malignant cells were positive for CD45, CD20, PAX-5, and MUM-1 while negative for CD3, CD10, CD138, and HHV-8 by IHC. IHC stains for BCL2, BCL6, and MYC protein were positive. Ki-67 was positive with a high proliferation rate (>80%). Microscopic images were examined at 400× objective.

Flow cytometry on the dissociated pleural biopsy failed to reveal a monoclonal B-cell population or an aberrant T-cell population. Culture on the pleural fluid failed to demonstrate any organisms. On cytology of the pleural fluid, large atypical lymphoid cells with irregular nuclei, prominent nucleoli, coarse chromatin, and a moderate amount of cytoplasm were seen. Some of the cells also exhibited plasmacytoid morphology. The malignant cells were positive for CD45, CD20, PAX-5, and MUM-1 while negative for CD3, CD10, CD138, and HHV-8 by immunohistochemistry. Stains for BCL6, BCL2, and MYC protein were positive. Ki-67 was positive with a high proliferation rate (>80%) (Figure 5). Flow cytometric analysis on the pleural fluid was not performed since no fresh cytology specimen was available to run the flow cytometry studies. Nevertheless, this is a limitation in our study, and we acknowledge that flow cytometry would have better characterized the cell composition, detailing the abnormal cell antigenic profile more extensively and highlighting the proportions of the various cell populations present in the fluid. In the absence of any evidence of lymphoma elsewhere and since the only site of disease was the pleural cavity, the diagnosis of fluid overload-associated large B-cell lymphoma (FO-LBCL) was made.

The patient was given wound care instructions along with physical therapy instructions. The patient will be observed and advised to follow up with a hematology-oncologist for his FO-LBCL.

## 3. Discussion

The WHO-HAEM5 differentiates FO-LBCL and primary effusion lymphoma (PEL) as two distinct entities [1]. Both typically present with an isolated effusion in the pleural, peritoneal, and/or pericardial cavities in the absence of an identifiable tumor mass [1]. PEL often presents in young to middle-aged immunocompromised patients and has a strong male predominance of 6:1 [6]. There have been rare cases of PEL described in elderly patients from regions in which HHV8 is endemic [7]. Given the absence of a standard treatment and its aggressive nature, the prognosis of PEL is poor, with a median survival of less than six months [8]. In contrast, FO-LBCL most commonly presents in elderly immunocompetent adults with a median age of 79 years with a slight male predominance (5:4) [9].

FO-LBCL presents as effusions with exclusive localization to body cavities, most commonly the pleura [10]. In a study by Gisriel et al. including 202 cases of HHV-8-negative effusion-based LBCL, fluid overload was reported in 56% of patients, mainly due to congestive heart failure (CHF), cirrhosis, and chronic renal failure [9,11]. Around 60% of the reported cases of FO-LBCL have been observed in Japanese individuals. Clinical outcomes for FO-LBCL are also largely favorable compared to PEL with effective aspiration only or with the addition of chemotherapy [12]. In a retrospective study of the clinicopathologic features and prognosis of FO-LBCL in Japan, the authors observed a 2-year overall survival rate of 84.7% [13]. These clear distinctions of prognosis highlight the importance of identifying these entities as distinct from one another [14].

Current working theories regarding the etiology and underlying pathogenesis of FO-LBCL have not been successful in determining a definitive cause [15]. In a case review, Alexanian et al. observed an HCV co-infection rate of 26.5%, significantly higher than the baseline HCV prevalence of 2% in the United States [5,16]. In their case series and literature review, Kobayashi et al. reported an observed HCV co-infection rate of 30–40% [17]. However, Kaji et al. only documented 1 HCV-positive patient out of the 64 patients analyzed in their Japan-exclusive retrospective study [13]. Ohshima et al., in their case series of five Japanese patients with HHV-8/HIV-negative PEL, uncovered genomic abnormalities and aberrations in all cases, postulating that multi-step genomic abnormalities may play a role in the development of FO-LBCL [18]. In addition to an underlying viral etiology or genomic abnormalities, FO-LBCL has also been postulated to be secondary to the effusion itself [5]. Detecting HCV-RNA in the peritoneal fluid of patients with FO-LBCL indicates that persistent antigenic stimulation may instigate a causative role in the pathogenesis of this lymphoma by promoting the clonal expansion of intraperitoneal B-cells [9,19].

In diffuse large B-cell lymphoma (DLBCL) associated with chronic inflammation, the setting of chronic inflammation in a site in which Epstein–Barr virus (EBV)-transformed B-cells are present results in these cells avoiding immune surveillance, thus providing them with the opportunity to become malignant cells [7]. In a similar manner, it has been proposed that chronic inflammation leading to cytokine dysregulation may be a predisposing factor to the development of FO-LBCL [20]. Ashihara et al. suggested that localized serositis may create a setting for malignant lymphoma to develop [21]. Given the presence of comorbidities predisposing patients to fluid-overloaded states in over half the cases they reviewed, Alexanian et al. believe that these findings support the possibility that FO-LBCL may be secondary to an underlying effusion [5]. However, they noted that no observations thus far have demonstrated a true causative association [5]. Interestingly, only 9% of the cases summarized by Gisriel et al. were positive for EBV, and the non-germinal center B-cell like (GCB) subtype accounted for 79% of cases [9]. In the same study, *BCL2*, *BCL6*, and *MYC* gene rearrangements were detected in 11%, 29%, and 19% of cases, respectively [9].

Cases presented by Kobayashi et al. revealed lymphoma cells which had anaplastic to immunoblastic morphology, with plasmacytoid features being present in some cases as well [17]. Interestingly, it is possible that the lymphoma cells in the effusions of patients with FO-LBCL tend to demonstrate terminal differentiation to plasmablastic or plasmacytic stages, in contrast to the conventional solid DLBCL [9]. Wu et al., in their case series and review, also revealed that most FO-LBCLs demonstrate anaplastic cellular morphology, with a small number of cases showing small- to medium-sized cells identified as Burkitt-like lymphoma cells [3]. Kaji et al. reported that all cases they included in their analysis displayed large cell centroblastic morphology. Kaji et al., Alexanian et al., and Kobayashi et al. reported that most of their reviewed cases (73–100%) expressed pan-B-cell antigens, such as CD19, CD20, or CD79a [5,13,17], as in our patient. Although one case reviewed by Alexanian et al. lacked pan-B-cell antigens entirely, the FO-LBCL did display gene rearrangement consistent with B-cells [5].

Flow cytometric analyses of reported cases reveal that a majority do demonstrate immunoglobulin light-chain restriction [13]. In our case, flow cytometric analysis was not performed on the pleural fluid. In 35 of the 64 cases analyzed by Kaji et al., a median Ki-67 proliferation index was 73.5% [13]. EBV-encoded small RNAs was also detected in 13–28.9% of cases studied by Kaji et al. and Alexanian et al. [5,13].

Conventional karyotyping of FO-LBCL overall appears to reveal a complex karyotype and copy number landscape with regions rich with focal copy number aberrations (CNAs) [2,9]. In the eight cases studied, Mendeville et al. utilized shallow whole-genome sequencing and targeting sequencing, measuring a mean of 33.6 CNAs per case [2]. The most repeated mutations occurred in *HIST1H1E* and *MYD88*, with many of them having a somatic hypermutation pattern. Other sites of recurrent mutation included *BTG1/2*, *IRF4*, *SYNE1*, *CREBBP*, *KMT2D*, and *MEF2B* [2]. The *MYC*, *BCL2*, and *BCL6* loci have been previously described in the literature as the most frequent sites for translocation [3,5,22,23]. While Mendeville et al. observed similar findings in their studied cases, they also identified *TP63*, *EXOC2*, and *KMT2D* as new translocations that had not been identified prior [2].

The main differential diagnosis of FO-LBCL includes PEL and DLBCL (Table 2). PEL mostly affects young to middle-aged, HIV-positive individuals, with a male predominance. PEL commonly exhibits plasmablastic cytology, which can at times also be seen in FO-LBCL. However, it is typically negative for pan-B-cell markers (CD20, CD79a, and PAX5) in contrast to FO-LBCL. HHV8 is consistently positive by definition in PEL, and EBV infection is noted in most cases. In contrast, FO-LBCL is usually seen in elderly patients and expresses pan-B-cell markers with no HHV8 infection and low association with EBV. Pyothorax-associated lymphoma (PAL) is a prototype of DLBCL associated with chronic inflammation, where lymphoma develops in the pleural cavity of patients with pyothorax. Patients with PAL are usually young and have a long history of chronic pyothorax or chronic pleuritis due to therapeutic artificial pneumothorax or tuberculous pleuritis [9]. In contrast to FO-LBCL and PEL, PAL presents with mass lesions in the pleura and/or lung near the pleura and is strongly associated with EBV infection [9]. It is important to note that high-grade B-cell or T-cell lymphomas can manifest as lymphomatous effusions, including Burkitt lymphoma, blastoid/pleomorphic mantle cell lymphoma, peripheral T-cell lymphoma, and anaplastic large-cell lymphoma, and it is crucial to exclude any other lymphomas before making the diagnosis of FO-LBCL.

Due to the limited number of cases, there is still no standard therapeutic regimen for FO-LBCL, and no clinical trials have been conducted yet. In the study from Kaji et al., most patients received immediate systemic chemotherapy as a first-line therapy, including CHOP or a CHOP-like regimen with or without rituximab [13].

## 4. Conclusions

The present study reports a case of fluid overload-associated large B-cell lymphoma (HHV8-negative effusion-based lymphoma) and presents a review of the clinical and histopathological characteristics of biologically similar cases reported in the literature. FO-LBCL mostly affects elderly and otherwise immunocompetent individuals. It is not associated with HHV8 infection, and EBV is mostly negative. Most cases are typically of non-germinal center B-cell like (GCB) subtype. Pan B-cell markers are always positive, which helps distinguish it from PEL. And no lymph node involvement is present, which is a major factor in differentiating it from DLBCL. FO-LBCL is associated with a favorable prognosis, and prognosis is largely determined by co-morbidity. The clinical behavior of this rare type of lymphoma is obscure, so a more extensive clinicopathological analysis of additional cases is needed to better understand this disease.

## Figures and Tables

**Table 1:** Relevant laboratory results of the patient.

Blood Test	Patient Value	Reference Range
White blood cell (WBC) count	7.89 × 10^3^/μL	4.8–10.8 × 10^3^/μL
Segmented neutrophils	43.3%	42–75%
Absolute neutrophil count	3.42 × 10^3^/μL	1.8–7.2 × 10^3^/μL
Red blood cell (RBC) count	6.3 × 10^6^/μL	3.93–5.22 × 10^6^/μL
Hemoglobin	11.1 g/dL	12.0–16.0 g/dL
Hematocrit	39.6%	37.0–47.0%
MCV	65.3 fL	79.0–92.2 fL
MCH	18.3 pg	25.6–32.2 pg
MCHC	28.0 g/dL	32.0–36.0 g/dL
Platelet count	65 × 10^3^/uL	150–450 × 10^3^/uL
Serum creatinine level	0.69 mg/dL	0.55–1.02 mg/dL
Blood urea nitrogen (BUN) level	8.0 mg/dL	7–18 mg/dL
Prothrombin time (PT)	16.1 s	12.4–15.2 s
INR	1.3	0.1–1.1
AP thromboplastin time (PTT)	34.9 s	24.7–39.8 s
pH	7.38	7.35–7.45
PCO_2_	42.0 mmHg	35.0–45.0 mmHg
PO_2_	91.0 mmHg	75.0–100.0 mmHg
HCO_3_	24.8 mmol/L	22.0–26.0 mmol/L

**Table 2:** Pathologic features differentiating FO-LBCL from PEL and DLBCL.

	FO-LBCL	PEL	DLBCL
Morphology	Variable morphology between large immunoblastic, plasmablastic, or anaplastic large cell lymphoma	Variable morphology between large immunoblastic, plasmablastic, or anaplastic large cell lymphoma	Diffuse sheets of large, atypical cells with vesicular chromatin and prominent nucleoli
Pan B-cell markers	Positive	Negative	Positive
EBV	+ve (13–30% [5,11])	+ve (80% [1])	+/− (<6% [1])
HHV-8	Negative	Positive	Negative
Lymph node involvement	No	No	Yes

## Data Availability

Not applicable.
